# The Treatment of Frost-Bite by Gomenol Oil Dressings

**Published:** 1918-02

**Authors:** 


***The Treatment of Frost-Bite by Gomenol Oil Dressings.*** By M. Alglave, reported by M. Arrou. Trans. and Abs. of a Series of Reports and Discussions in the *Bulletins et Mémoires de la Société de Chirurgie de Paris,* Feb. 16, March 2, March 9, March 16, and April 27, 1915.

A. practiced conservative treatment upon 23 cases of frost-bite of varying degrees of seriousness. 9 of the cases would otherwise have called for amputation.

Instead, A. washed the feet with tepid water and soap, and then with ether. When the skin was completely dried, compresses saturated with gomenol oil (1 : 10) were applied, and protective wads of cotton placed over them. Astonishing results were obtained. Although two patients lost toes, and one a large section of gangrenous flesh, all were ultimately cured without recourse to amputation. Another case, complicated with tetanus, could not be saved.

M. P. Sebileau then questioned even the necessity for the use of gomenol oil, and stated that in several serious cases he had succeeded by merely applying wads of cotton and hot water bottles. Later the skin was dried with warm air.

M. L. Labbé also believed that amputation was unnecessary in serious cases of frost-bite.

M. E. Quénu believed that treatment should vary accordingly as the cases were superficial or accompanied with mortification of deeper tissues. As soon as the drying and elimination of tissues extended below the skin, the case was no longer one of freezing, but one of dry gangrene resulting from freezing, with corresponding indications for operation.

The operation should be performed as soon as it is clear that the line of elimination has gone below the skin, but not until all edema of the foot has disappeared. The operation can usually be carried out at the end of 3 or 4 weeks, and is necessary to prevent sepsis and ascending neuritis.

M. Tuffier believed that in most cases of trench feet it is better not to envelop the member in warm cotton, which tends to increase the pain, but, after cleansing, to leave it exposed or covered with a thin ointment of oil. It is then best to wait and, in any case, not to hurry amputation. He mentioned one instance in which the skin indicated mortification as high as the ankles, but which, after a few days, lost most of the signs. Even if there is a line of demarcation, it is not necessary to operate at once. It is necessary to amputate only when moist gangrene complicated with sepsis appears. Often, however, these symptoms of sepsis may be overcome by *douches* of hot air. Thus moist gangrene may sometimes be converted into dry gangrene.

T. then proceeded to speak more especially of *trench feet* as an individual malady. It results from disturbances of the circulation when there is no snow, ice, or frost. Within 2 months he had observed 200 cases. In the Vth Army from the 13 to the 21 of November the previous year, out of 16 000 men there were 678 cases of trench feet. Proper preventive measures, however, soon greatly reduced this incidence.

When a man came to the service he would usually state that he had suffered for about 2 days from a painful numbness of the feet, especially on one side of them. As soon as he removed his boots, the feet swelled, turned white, and this appearance extended half way up the leg. Often the skin would be anesthetic. Sometimes the numbness interfered with walking. In light cases the whiteness gave place to a diffuse reddishness, and all symptoms disappeared in the course of from 24 to 48 hours. 238 cases out of 659 were of this nature. 270 others, while not serious, were more severe. In these a phlegmon of the feet followed the edema. Blisters appeared on the dorsal surface of the foot sometimes the size of a mandarine; and these were often so painful as to prevent sleep. The toes would probably turn black and become insensible. In such cases the circulation would reestablish itself gradually; or black spots of gangrene might appear, generally on the plantar face of the toes, and slowly eliminate themselves like a dry gangrene. For a long time the feet would remain painful.

In the very few serious cases, the whole toe and a part of the metatarsus would become gangrenous as is the case in instances of genuine freezing.

One of the peculiarities of the disease was that it occurred when the temperature was well above freezing. It resulted almost always from the same conditions : the men had been in the front trenches of the first or second line. Usually these had been flooded with water, but the water had not been frozen, nor the temperature at any time below freezing. Almost always the men with this complaint came from the same trenches, and had been standing ankle-deep from 4 to 7 days in muddy water without removing their shoes. In carefully kept trenches, on the contrary, those in which the water had been properly drained off and wooden flooring laid down, no cases had been found.

Other conditions contribute, such as the prolonged squatting posture, long periods in the trenches without relief, and especially the pressure on the feet and legs of puttees or lacings. All of these last conditions, however, are present in the duties of sentinels and artillery men, who never develop trench feet. It is, therefore, most probable that cold is the essential cause.

The *Service de Santé* by way of prevention distributed vaseline. This was later replaced by lanoline, which proved distinctly better. The feet were greased each day, and thus the shoes had to be unlaced. The trenches were properly drained, and the men urged to exercise their toes and feet as much as possible. More frequent shifts were arranged. In the active service, however, all these precautions are not always possible.

MM. Quénu and Mauclaire expressed the belief that tight wrappings on the legs and feet exerted an important influence, although the latter had known several serious cases in which this factor could be entirely eliminated. He also stated that he believed that amputation was often necessary. The cases he had in mind, however, were genuine cases of freezing.

M. P. Sebileau in reply stated that he believed that amputations were in some cases necessary, but that this was a late consideration. It was never necessary, he believed, to amputate primarily because of the appearance of freezing. He furthermore saw no reason to distinguish, as did M. Tuffier, between actual freezing and the condition known as "trench feet" when the temperature has not fallen below freezing. Anatomically and etiologically he believed the conditions were the same, and that the treatment should be the same. The difference was not one of kind but merely of degree.

M. Kirmisson stated that there was a real difference between frozen feet and trench feet, if not from a theoretical point of view, at least from a practical one. It had long been known that a lowered temperature combined with dampness and constriction of the feet and limbs produced a devitalizing effect upon the tissues; and this was precisely what happened in the case of the men in the trenches. The difference between this malady and actual freezing was of vital importance from a therapeutic point of view.

M. Walther cited two serious cases of trench feet, one with complete grangene of three toes, and partial gangrene of two. The other had gangrene of almost the whole foot, with a triangular flap of vital skin remaining on the inside of the foot. No operation was performed. The elimination of the devitalized tissues was greatly facilitated by the use of lacteol powder on the dressings. In one case after the elimination, the toes were almost completely detached and could easily be removed with scissors. After the healing was complete a slight regularization was practiced. Neither of these patients had worn puttees, and neither had been subjected to a temperature below freezing, but merely to cold water for a prolonged period.

M. Paul Reynier then emphasized the inaction, which, he believed, next to cold itself, was the most important factor in producing trench feet. He stated that although the soldiers in the war of 1870 were subjected to greater cold than those in the present war, and although they wore tightly laced shoes, they had fewer cases of frozen feet than the present fighting force because they were more constantly on the march and were not subjected to the inactivity of the trenches. He called attention, also, to the marked differences of susceptibility in different people, and at different ages.

M. Picqué called attention to the fact, known since 1812, that the greatest number of frozen feet was to be noted among armies during a thaw.

M. Quénu protested that M. Walther's advise to postpone operation until spontaneous elimination had occurred, might lead to serious complications, especially neuritis.

M. Tuffier insisted again upon the distinction he had drawn between actual freezing and trench feet. The effects of freezing by ice and snow had long been known to all, but the occurrence of trench feet had greatly disconcerted the whole medical profession. As to the degree of temperature, he did not believe that any one present knew of a case of gangrene due only to cold, when the temperature had not fallen below freezing.

M. Souligoux reported at considerable length a case of genuine freezing, due to exposure on the battle-field. Both feet were affected, but the right was much the worse. This foot was swollen and white, but there were no blisters. The leg also was swollen. The skin was taut and, when pressed, retained the mark of the finger. In the case of the left limb the symptoms were confined to the foot itself. On the fourth day blisters appeared on the plantar arches of both feet, and fever set in. The blisters contained a reddish serum. They were pierced and aseptically bandaged. The sense of touch was considerably reduced but not entirely destroyed. On the ninth day fluctuation was noticed on the outer side of the right leg above the ankle. An incision permitted the escape of a white serosity. As the temperature continued to rise, 2 days later an incision was made still higher among the peroneal muscles, and these were found to be infiltrated with the serum. This relief caused the temperature to fall from $40.5^0$ to $38^0$. It seemed as if the foot might be saved.

Shortly, however, new symptoms appeared. On the inside middle face of both feet gangrene was observed. Necrosed tissues composed of the plantar aponeuroses and tendons began to be eliminated. The temperature rose again to $40.5^0$. Under chloroform, a careful examination led to the discovery that although the skin for the most part appeared healthy, the deeper tissues of the toes, of the plantar surface, and of the leg as far as the knee, were necrosed. This was true only of the right foot. The left, except for gangrene on the inside face, was in a satisfactory condition.

The right leg was amputated above the knee.

The case was chiefly valuable because it proved that while the skin appeared reasonably healthy, the tissues under it might be completely necrosed from the cold.

M. Walther again raised the question as to when it was necessary to operate. He recognized that if actual symptoms of neuritis were present it would be better to amputate, but otherwise he had found it best to wait at least until the necrosed parts had been spontaneously eliminated.

M. Paul Thiéry, after referring to cold, compression, and inactivity as the principal factors in producing trench feet, declared that not enough attention had been paid to individual differences in resistance. Lymphatic and scrofulous persons offered a very weak resistence. Hyperidrosis, common among soldiers even in winter, was another predisposing condition calling for treatment.

The speaker stated that in treating 14 cases of frozen toes he had not been obliged to amputate once. He then outlined his method of treatment :

Upon the arrival of the patient, he gave a warm foot bath with soap to remove grease, and, to complete the cleansing, the toes were rubbed with alcohol heated to 90°. He was next given a foot bath in a cold saturated solution of picric acid (about 12 : 1,000). In this manner a sort of tanning took place, and almost immediately the pains ceased. The toes healed rapidly. In from 6 to 12 days the patients could leave the hospital. Such treatment was cheap and effective, but should be used only for light cases. It would be useless in cases of total freezing, in which whole toes become gangrenous.

T. thought that the picric acid treatment should be given prophylactically against hyperidrosis. Twenty years before, he had shown that foot-baths of this acid prevented sweating of the feet, and he knew of at least one case in which a group of men in the trenches found that it prevented them from having trench feet.

M. Arrou, summarizing the points already made, declared that no one could deny that a gangrenous condition could be produced in man by a temperature that had not fallen to the freezing point. This was recognized in the Russo-Japanese war. As to the cause, it was likewise certain that compression in any form was dangerous. This had long been recognized by arctic explorers. It was likewise obvious that inactivity was a contributing cause. Less certain, though probable, were the personal factors mentioned. It should not be forgotten, however, that cold was the one invariable condition, and in itself was all-sufficient to produce the phenomena in question.

As to treatment, A. believed that the gomenol oil (1 : 10) recommended by M. Alglave was most effective. He had found a similar preparation, pyroleol, most efficient in relieving burns. Upon the disputed point of operative treatment, A. believed that amputation should not be delayed until secondary neuritis set in. On the other hand, it was certain that operations should not be undertaken too soon. In this respect most surgeons erred. It was decidedly the best policy to wait until nature had been given a chance to effect her own cures, and by that time it was often found that no amputation was necessary. Thus by waiting M. Alglave had saved all of his 23 cases.

M. H. Hartmann reported observations made at the front by MM. Bressol and Desgouttes. These workers had noted no cases of freezing of the nose, ears, or upper members. They described three types, or rather stages, of the condition of the lower members :

1. Only redness of the skin is noticed in the first. This may be diffused or in separate blotches, extending upwards for various distances. Anesthesia, more or less complete, is an attending symptom. Spontaneous pains are felt, especially at night, or when there is increased heating. The red blotches, sometimes of a violet tint, are usually to be found on the dorsal surface of the foot, or at its outer edge, at the head of the first metatarsus, or at the toe joints.

2. In the second stage there is edema, especially of the dorsum and of the toes. The skin is rarely of a normal color, but there is no blistering or ulceration.

3. The third stage is marked by blisters and ulcerating or gangrenous patches. The blisters, like those of burns, usually contain a yellowish serum when they are on the edematous dorsal surface. On the plantar surface, however, they are usually larger and filled with a reddish liquid. Localized ulcers also may appear at the points where the shoe binds, on the dorsum of the foot, at the head of the first metatarsus, and sometimes on the toe joints. These ulcers are quickly infected, and give the appearance of moist gangrene.

In some cases there is a generalized gangrene of the arterial form. In most serious cases the line of demarcation is at the point of Syme's incision. The gangrene is always more extensive on the dorsum of the foot than on the plantar surface where the blood supply is greater.

B. and D. did not agree with those who considered tight shoes and leggings the chief cause of trench feet. They believed that *cold*, and especially *damp cold*, was chiefly responsible.

Most of the patients had been exposed in the first trenches for from 4 to 7 days. When the shifts were more frequent, the incidence was greatly reduced. The first trenches were generally narrow and shallow and badly drained. To avoid detection by the enemy, the men had to bend over, and the hypertension on the knee constituted a serious predisposing condition. Often when a man crouched with one leg bent and the other extended, the symptoms appeared chiefly or only in the foot of the leg that was bent.

B. and D. declared that they had noticed many more cases of trench feet when the men had to stand in water or mud at a temperature slightly above freezing, than when the temperature was much lower and the ground completely frozen. Larrey, a century ago, made clear that cold combined with humidity was a more important factor than dry cold. Thus it happened that no cases of frozen noses, ears, and hands where reported in the present war.

Although pressure of shoes and leggings might sometimes render the feet more liable to the action of the cold and damp, this pressure could not be considered by any means the determining cause. It was true, however, that locally the binding of the shoes might be important in causing localized blisters and gangrene, especially on the dorsum of the foot, on the joints of the toes, and on the first metatarsus. Thus it happened that in moderate cases, local pressure was often an important cause of gangrene, whereas the cold produced the edema. Soldiers often stated that their shoes were loose enough until the swelling of the feet began. It was therefore probable that the tightness of the shoes at first was not a causal factor at all. As evidence that puttees were not responsible, B. and D. had noticed many cases in certain regiments that did not wear puttees at all. Furthermore, no cases were reported until December, when the period of cold and rain began. Surely the more serious cases of total gangrene were the result of long exposure in humid cold or to very low temperatures of dry cold.

B. and D. declared themselves much surprised to hear that in the front trenches the soldiers had been ordered to remove their shoes and puttees, and that in consequence the incidence of trench feet had fallen from 30 o/o to 3 o/o. They doubted that in the first line trenches this sort of relaxation would ever be officially permitted. Perhaps some of the trenches might be improved by brush flooring, but from what they had themselves seen of the difficulties encountered in actual war by the occupants of these trenches, they saw little hope of any marked improvement of this kind.

M. Tuffier reported a case that had come to his attention, of a patient who had been wounded and had remained 12 hours with his arm resting in water considerably above freezing and even mild in temperature. Although his chest wound was then almost healed, he was still suffering from the freezing of the arm that had remained in the water.

M. Phocas expressed himself as convinced by his experience in Greece that *prolonged inactivity* and *humid cold* were the chief factors in producing trench feet. Among the Greek troups cases were most frequent among those stationed in advanced posts where they were wholly inactive. They occurred not so much in extremely cold weather as in damp conditions. He had not found the symptoms always the same as those noted in cases of actual freezing. No prickly sensation of the skin was ever mentioned.

The speaker called special attention to the influence of a filthy condition of the feet upon the prognosis of the disease. From this source proceeded most of the infections that turned dry gangrene into moist gangrene and made amputation necessary. He agreed with M. Sebileau that primary amputation was never indicated, and that it sufficed to disinfect the feet and then remove the case to a base hospital for further treatment. The question of amputation rarely arose until the line of demarcation clearly showed what portions of the foot and toes must be sacrificed. The speaker believed that it was then preferable to amputate a little behind the line of demarcation rather than to wait until spontaneous elimination had taken place. By such an operation the danger of complications were avoided and a considerable economy of time realized.

He cited several cases in which, because of complications, amputations had to be performed before the line of demarcation appeared; but he stated that such occurrences were rare. He expressed little faith in any form of therapeutic dressing, and believed that carefully aseptic bandaging was all the treatment needed.

M. H. Hartmann reviewed briefly two reports, one made by M. Berthélemy and the other by MM. Orticoni and Delage, covering 4,657 cases. All three concluded that compression caused by shoes or leg bands was only a secondary factor in producing trench feet, and that it was often absent entirely. B. believed that inactivity in damp trenches was the chief predisposing condition, especially if the men were crouching. In this position, often half asleep, they were easily victimized. The officers, on the other hand, who were obliged to change their positions frequently were rarely affected. B. recommended that the men should be made to move about more during their two-day shifts in the trenches. O. and D. believed that if rubber shoes were worn outside of felt ones, and the feet greased, cases of trench feet could be avoided.

O. and D. recommended by way of treatment the thorough desinfection of the feet, and bandaging with antiseptic pommades or substances like picric acid. They insisted that the patient should be warmed, but not the lesion. It had been found best for the limb to be raised. The patients found it most comfortable to lie on their stomachs with the affected limb bent perpendicular to the bed, and left free to move. This movement was important in hastening the cure, and was a great relief to the patient.

